# The Coaches’ Perceptions and Experience Implementing a Long-Term Athletic Development Model in Competitive Swimming

**DOI:** 10.3389/fpsyg.2021.685584

**Published:** 2021-05-28

**Authors:** Mário J. Costa, Daniel A. Marinho, Catarina C. Santos, Luís Quinta-Nova, Aldo M. Costa, António J. Silva, Tiago M. Barbosa

**Affiliations:** ^1^Department of Sport Sciences, Polytechnic Institute of Guarda, Guarda, Portugal; ^2^Research Centre in Sport Sciences, Health Sciences and Human Development, CIDESD, Vila Real, Portugal; ^3^Department of Sport Sciences, University of Beira-Interior, Covilhã, Portugal; ^4^Department of Sport Sciences, Polytechnic Institute of Castelo Branco, Castelo Branco, Portugal; ^5^Department of Sport Sciences, Exercise and Health, University of Trás-os-Montes and Alto Douro, Vila Real, Portugal; ^6^Department of Sport Sciences, Polytechnic Institute of Bragança, Bragança, Portugal

**Keywords:** training, swimmers, long-term planning, career, coaching

## Abstract

The aim of this study was to analyze the association between coaches’ experience and their perceptions on the implementation of a long-term athletic development (LTAD) model created in 2016 by the Portuguese Swimming Federation. Eighty-six swimming coaches were assembled in groups according to their experience level: “novice” (*n* = 24), “intermediate” (*n* = 26), and “experienced” (*n* = 36), and they answered a questionnaire with the following items: (i) awareness of the existing model (ii) acceptance (iii) usefulness for practice, and (iv) implementation of this model by their peers. Regardless of experience, ~67% of the coaches were aware of the model. Among those, a large number showed acceptance (~95%) and confidence in its usefulness (~83%) for their daily practice. Most coaches (92%) showed concerns about the fact that their peers do not respect the model frameworks, declaring the search for their swimmers’ immediate success (~58%) as the main cause for such behavior. The results also showed an association between experience and knowledge about the model’s existence [*χ*^2^ (2) = 10.223, *p* < 0.01, *V* = 0.345], and experienced coaches exhibited better knowledge than their intermediate [*χ*^2^ (2) = 9.555, *p* < 0.01, *V* = 0.393] or novice [*χ*^2^ (2) = 5.926, *p* = 0.02, *V* = 0.314] counterparts. While there was an association between the coaches’ experience and knowledge about the LTAD model’s existence, this situation does not seem to influence the way coaches accept and understand the usefulness of the model for their daily practice.

## Introduction

The national recognition of a sport and the expandable governmental funding to support elite training depends on the international outcome (Hogan and Norton, 2000). Under this context, the development of strategies that would trigger larger sports participation or help to achieve a smooth progressive path to expertise is welcome. The Long-Term Athletic Development (LTAD) models have been considered one backcourt strategy to work with. LTAD models are known to have a set of principles that define general frameworks from a National Sports Federation and may help athletes to reach longer and more sustainable careers. Most of the LTAD models are similar in the way they present the general background to develop the important aspects of training, but they also differ in specific frameworks. While early models are classified as “general,” showing how youths are physically developed through maturation, the subsequent models provide more detailed information on resistance work or fitness-specific training methods (Pichardo et al., 2018). Nowadays, these models complement each other and work toward defining a more precise model for each sport.

Regarding this purpose, the Portuguese Swimming Federation created an LTAD model back in 2016 (Costa et al., 2016). A set of competencies (technical, physical, water‐ or land-oriented, psychological, tactical, recovering, competitive, parental counseling, training control/evaluation, and coaches’ skills) was drafted to serve as the main frameworks (Costa et al., 2016). However, no attempt was made to understand the coaches’ view on model suitability and the way they combined those principles into practice through the last couple of years. To date, one single study tried to understand how practitioners interpreted an LTAD model and implemented it locally (i.e., English swimming model known as “The Swimmer Pathway”). While there were some concerns related to regulations in amateur and age group competitions, the most disturbing aspect expressed by the participants was the impact of excessive training volume upon technical development at younger ages (Lang and Light, 2010).

While this type of inquiry is a good way to increase the coaches’ role in a national strategy, the perceptions may be dependent on the coaches’ experience or professional background (Mesquita et al., 2010). Coaches’ experience is considered the major underlying factor for the athletes’ development (Nelson and Cushion, 2006). Previous reports suggested that a minimum of 10 years of coaching is necessary to reach expertise (Abraham et al., 2006). For this reason, less experienced coaches perceive themselves as less competent than highly experienced coaches at some point (Santos et al., 2010). Additionally, the importance given to several training contents seems to differ among novice, intermediate, and experienced coaches (Leite et al., 2011; Serrano et al., 2013). Thus, the way coaches become aware, perceive, and implement certain strategic projects created by national sports federations may also be influenced by their experience.

In this sense, the purpose of this study was to analyze the association between coaches’ experience and their perceptions on the implementation of a LTAD model for swimmers. It was hypothesized that there is a significant association between the coaches’ experience and their perceptions about the LTAD model implementation.

## Materials and Methods

### Participants

A cross-sectional research design was employed. An electronic survey was applied to check the coaches’ availability to take part in the project. Eighty-six Portuguese swimming coaches (69 men and 17 women) volunteered to participate. The following inclusion criteria were defined: (i) holding a professional certificate by the national certification program and (ii) should be in charge of a group of swimmers affiliated in the Portuguese Swimming Federation. The coaches were assigned in three different level groups depending on the number of years of experience: (i) novice (up to 3 years) (ii) intermediate (from 4 to 9 years), and (iii) experienced (10 years or more). There was also an attempt to group the coaches by certification level and the development stage of their swimmers. Here the frameworks proposed by Balyi and Hamilton (2004) were used. Table 1 shows the distribution of the participants across the three groups and their basic characteristics. The University Ethics Committee approved the study design, and procedures were applied according to the Declaration of Helsinki.

**Table 1:** Basic characteristics of the swimming coaches by level of experience.

Experience	*n*	Age (yo)	Certification (*n*)	Athletes’ stage (*n*)
Novice	24	24.3 ± 3.7 yo	Level 1 (19)	Fundamentals (11)
			Level 2 (5)	Learning to train (9)
				Train to compete (4)
Intermediate	26	33.1 ± 5.4 yo	Level 1 (13)	Fundamentals (5)
			Level 2 (9)	Learning to train (11)
			Level 3 (4)	Train to compete (5)
				Train to win (5)
Experienced	36	41.9 ± 10.0 yo	Level 1 (10)	Fundamentals (11)
			Level 2 (13)	Learning to train (7)
			Level 3 (10)	Train to compete (8)
			Level 4 (3)	Train to win (10)

yo, years old; *n*, number of participants.

### Procedures

The coaches’ perceptions were assessed through a survey. The validity was checked by high-level and experienced swimming coaches, presenting both academic (at least a degree in Sports Sciences) and technical education (at least a coach certification of level 3). A preliminary version drafted by the authors was sent to three experienced coaches for them to check it terms of clarity and understanding. Consequently, two of them suggested minor accuracy corrections, mostly related to the last sub-section. Thus, minor edits were made after their examination, and the final version was designed for application.

The questionnaire had two distinct sections: (i) coaches’ biographic information and (ii) LTAD model. The biographic information gathered sub-sections such as age, years of experience, level of certification, and the stage of development of their athletes. The section about the LTAD model presented the following sub-sections (items): (i) knowledge of the current model (ii) acceptance (iii) usefulness, and (iv) implementation of the model by their peers. The questions were answered through a “yes” or “no” option. At the end, the “implementation” sub-section included two additional questions about the way coaches perceived their peers’ implementation of the model. If the “no” answer was chosen regarding other coaches’ lack of respect for the model frameworks, the following closed options were available: (i) showing ignorance of the model’s existence (ii) having ideological divergence about several aspects of the model (iii) club’s policy or pressure, or (iv) searching for the immediate success of their swimmers. This would help to understand why some coaches fail to apply the proposed frameworks and develop further strategies accordingly.

The questionnaire was applied individually in a quiet environment and took up to 15 min to fill out. In order to assess the reliability of the coaches’ perceptions, the temporal stability of measures was tested in 10% of the sample as reported in a previous study (Leite et al., 2011). A few coaches (*n* = 8) repeated the procedure 2 weeks later under the same conditions, showing 95% of agreement.

### Statistical Analysis

Exploratory data analysis was used to identify potential outliers. Data was treated in terms of mean and SD for each group when necessary. Frequency and percentage count were used for the “yes” or “no” options according to the coaches’ experience. Pearson’s chi-square test (*χ*^2^) was used to establish whether the coaches’ experience was associated with their perceptions about the implementation of the LTAD model. A *post hoc* with multiple pairwise comparisons in 2 × 2 contingency tables was considered for the significant variables. The Cramér’s *V* (*V*) test was used as an effect size (strength of association) for the Pearson’s chi-square test and classified as follows (Cohen, 1988): very weak (0.1 < *V*), weak (0.1 ≥ *V* < 0.3), moderate (0.3 ≥ *V* < 0.5), and strong association (*V* ≥ 0.5). All statistical treatments were conducted using SPSS software, release 27 (IBM, SPSS Inc., Chicago, IL, United States), and the significant cutoff value was set at *p* ≤ 0.05.

## Results

Among the 86 coaches inquired, only 58 (~67%) were aware of the model. A large number showed acceptance (~95%) and recognized the usefulness (~83%) of the LTAD for their daily practice. Frequency and percentage according to the coaches’ experience and perceptions are shown in Table 2. A large percentage of the experienced group answered the “yes” option for the “knowledge” (36.0%), “acceptance” (50.0%), and “usefulness” (41.4%). All groups presented a large percentage in the “no” option for “implementation” from their peers. In this field, a large number of coaches (92%) answered that their peers do not implement the Portuguese model. The main cause for this answer was the “search for the immediate success of their swimmers” (58%), followed by “showing ignorance of the model’s existence” (14%), “clubs’ policy and pressure” (12%), and “having ideological divergence about some aspects of the model” (9%); however, 7% of the coaches did not answer this question. Pearson’s chi-square test was significant in the 2 (“knowledge”) × 3 (coaches experience) analysis and had a moderate association. The “acceptance,” “usefulness,” and “implementation from their peers” items were not associated with the coaches’ experience.

**Table 2:** Frequency, percentage, and statistical analysis according to the coaches’ experience and perceptions (items).

Items	Coaches’ experience			*χ*^2^ (*df*)	*p*	*V*
	Novice	Intermediate	Experienced			
Knowledge (*n* = 86)						
Yes	14 (16.3%)	13 (15.1%)	31 (36.0%)	10.223 (2)	0.006	0.345
No	10 (11.6%)	13 (15.1%)	5 (5.8%)			
Acceptance (*n* = 58)						
Yes	13 (22.4%)	13 (22.4%)	29 (50.0%)	1.804 (2)	0.406	0.176
No	1 (1.7%)	0 (0%)	2 (3.5%)			
Usefulness (*n* = 58)						
Yes	13 (22.4%)	11 (19.0%)	24 (41.4%)	1.651 (2)	0.438	0.169
No	1 (1.7%)	2 (3.5%)	7 (12.1%)			
Implementation (*n* = 86)						
Yes	0 (0.0%)	3 (3.5%)	4 (4.7%)	3.742 (2)	0.154	0.254
No	24 (27.9%)	23 (26.7%)	32 (37.2%)			

%, percentage; *χ*^2^, Pearson chi-square test; *df*, degrees of freedom; *V*, Cramér’s *V*; *n*, number of participants.

The *post hoc* for “knowledge” noted an intergroup difference (Table 3). A significant association between the novice and experienced groups was observed as well as between the intermediate and experienced groups. However, no association was found between the novice and the intermediate.

**Table 3:** Frequency, percentage, and statistical analysis according to the “knowledge” item.

Experience	Knowledge (*n* = 86)	*n* (%)	*χ*^2^ (*df*)	*p*	*V*
Novice (N) – intermediate (I)	Yes	N: 14 (51.9)	0.349 (1)	0.555	0.084
		I: 13 (48.1)			
	No	N: 14 (51.9)			
		I: 13 (48.1)			
Novice (N) – experienced (E)	Yes	N: 14 (31.1)	5.926 (1)	0.015	0.314
		E: 31 (68.9)			
	No	N: 10 (66.7)			
		E: 5 (33.3)			
Intermediate (I) – experienced (E)	Yes	I: 13 (29.5)	9.555 (1)	0.002	0.393
		E: 31 (70.5)			
	No	I: 13 (72.2)			
		E: 5 (27.8)			

%, percentage; *χ*^2^, Pearson chi-square test; *df*, degrees of freedom; *V*, Cramér’s *V*; *n*, number of participants.

## Discussion

The aim of this study was to analyze the association between the coaches’ experience and their perceptions on the implementation of the LTAD model for swimmers. The results confirmed that experienced coaches tend to have more knowledge about the model’s existence than their peers. Despite that, the experience seems not to influence the way coaches accept and understand the usefulness of the model for their daily practice.

Regarding the basic characteristics of the coaches included in this study, some important aspects should be highlighted. In this sample, the coaches with less experience are the ones responsible for the development programs, which is in line with previous studies (e.g., Leite et al., 2011). It is consensual that the initial stages of development are the most sensitive for learning the fundamental skills necessary for each sport (Balyi and Hamilton, 2004; Pangrazi and Beighle, 2016; Haibach-Beach et al., 2018). Although some argue that experienced coaches need to be stimulated to take part in the swimmer’s initial development, this aspect is yet to be accomplished. Another element to be considered is the large number of experienced coaches that do not yet hold the final levels of certification. The way the coaching education system is organized in Portugal can help to explain this anomaly. Some degree of dissatisfaction still exists regarding the current education framework in Portugal, which remains excessively theoretical within classrooms (Mesquita et al., 2014). Generally, coaches believe that daily work with experts or peers (Abraham et al., 2006) and learning by doing (Erickson et al., 2008) are more friendly sources of knowledge than being in formal training settings. With this in mind, several coaches avoid pursuing additional certification levels to seek new goals and knowledge. Nevertheless, further actions should be taken whether by the government or national federations since minimal professional certification is required to train each stage of the swimmer’s development.

The results also showed that most of the coaches are still unfamiliar with the proposed model. The LTAD model is part of the national certification program for swimming coaches (level 2) curriculum only since 2016. As part of the certification course level 2, this is not developed at the initial stages of formation (level 1) or at the latter ones (levels 3 or 4). This can explain why several coaches from lower to higher levels did not have access to this model. As previously observed, the way coaches gather knowledge comes from a broad range of sources and not necessarily by participating in the national coaching certification programs (Mesquita et al., 2010). Although the Portuguese Swimming Federation has dedicated large efforts to promote this model through several media sources, the effort does not seem to be enough to reach out to most coaches. Therefore, more effective dissemination strategies should be employed to overcome this constraint.

The coaches’ level of experience seems to play an important role on the awareness of the model. There was an intergroup difference that showed that experienced coaches tended to have more knowledge about the model’s existence than the novice or intermediate ones. Based on cognitive psychology, experts are able to pursue and apply knowledge in a more professional manner to solve complex problems in a specific domain (Abraham and Collins, 1998), so we may argue that more experienced coaches are able to look further and search deeper for information that may complement their training plan. In spite of the awareness of the model’s existence, experience seems not to determine the way coaches accept and see model usefulness. In fact, coaches who possess different certification levels have already shown similar concepts about the importance attributed to knowledge sources (Mesquita et al., 2010). Despite that, the Portuguese Swimming Federation made every effort to create a model attending to overall aspects that define the swimmers’ development. A benchmark strategy was employed gathering and adapting information already written in LTAD models used in other countries. Additionally, several aspects deemed important for performance (Barbosa et al., 2013) were also explored. Thus, a step further would be to understand how coaches perceive the importance of each framework presented in the model and adjust the LTAD model accordingly.

An interesting result was related to the implementation of the model. Most of the coaches showed concerns about their peers not respecting the frameworks. Coaches claim that their peers search for the short-term success of their swimmers. This outcome confirms previous findings (Lang and Light, 2010) about practices in England after the LTAD model implementation. It is consensual that technical work should be the focus at the initial stages of the swimmers’ development (Nugent et al., 2017). Meanwhile, some coaches compromise or neglect this kind of work at younger ages, mostly inducing larger training volumes. We may speculate that this happens mostly for personal ascension or to join better squads. Nevertheless, this topic still remains an alarming fact and should be emphasized in the near future.

Few limitations should be considered for further interventions: (i) the small sample size did not allow us to explore deeply perceptions by gender or other kinds of cohorts and (ii) the experience thresholds used for grouping were defined on the basis of both empirical and scientific data.

In summary, this study presents a new way to assess how the sports policies implemented by the National Sports Federations reach end-users in the field, such as coaches. Coaches are given a more important role in a process that, along with the National Swimming Federation, can enhance youth engagement throughout their sports careers and increase the chance to reach an elite level. Further actions should be taken in order to reach the desired result. A national tracking system can be developed with the aim to match “certification levels” × “athletes group” and to monitor if every coach has the minimum certification level to be in charge of a specific group at the beginning of the season. The Portuguese Swimming Federation can use regular competitions to promote the LTAD model closer to the less experienced coaches. A new detailed enquiry is needed to understand the coaches’ perceptions about the different frameworks of the model in order to change it, if necessary.

## Data Availability Statement

The raw data supporting the conclusions of this article will be made available by the authors, without undue reservation.

## Ethics Statement

The studies involving human participants were reviewed and approved by Ethics Commity of The University of Beira Interior. The patients/participants provided their written informed consent to participate in this study.

## Author Contributions

MC, DM, AC, and TB conceived and designed the experiment. MC, LQ-N, and AS performed the experiment. CS, LQ-N, and AS analyzed the data. MC, DM, CS, AC, and TB drafted the manuscript. All authors contributed to the article and approved the submitted version.

### Conflict of Interest

The authors declare that the research was conducted in the absence of any commercial or financial relationships that could be construed as a potential conflict of interest.
